# Bibliometric mapping of artificial intelligence research in surgical education (1997–2025)

**DOI:** 10.3389/fsurg.2026.1759439

**Published:** 2026-03-10

**Authors:** Jinlin Wu, Junfei Zhao

**Affiliations:** Department of Cardiac Surgery, Guangdong Provincial People’s Hospital (Guangdong Academy of Medical Sciences), Southern Medical University, Guangzhou, China

**Keywords:** AI, artificial intelligence, bibliometrics, surgery, surgical education

## Abstract

**Background:**

Artificial intelligence (AI) is rapidly transforming surgical education, yet comprehensive analysis of research trends in this field remains limited.

**Methods:**

We analyzed publications from the Web of Science Core Collection using “surgery” AND “education” AND “artificial intelligence” as search terms (1997–2025). Bibliometric indicators were analyzed using the bibliometrix package in R.

**Results:**

We identified 572 publications by 3,228 authors across 332 journals, with an 18.39% annual growth rate. The United States and United Kingdom led research output, with Harvard University as the top contributing institution. “Augmented reality”, “video”, and “performance” emerged as mature research themes (motor themes), while large language models represent recent emerging topics. International collaboration accounted for 25.17% of publications, predominantly among developed nations. Citation analysis revealed human-robot interaction and AI-based simulation training as the most influential research topics.

**Conclusions:**

AI research in surgical education shows rapid growth but significant geographic disparities exist. Future efforts should focus on developing personalized learning systems and addressing the global digital divide in AI-enhanced surgical education.

## Introduction

In the rapidly evolving landscape of surgical education, the integration of advanced technologies is increasingly becoming a crucial factor for future surgical training (1, 2). Among these, artificial intelligence (AI) has emerged as a transformative force, demonstrating immense potential in enhancing surgical skills education, acquisition, and refinement processes (3, 4).

Traditionally, surgical education has primarily relied on the apprenticeship model, where surgical residents learn under the guidance of experienced surgeons through direct observation and practical operations. However, this model presents several limitations, including inconsistent teaching quality, limited exposure to diverse surgical cases, and difficulties in providing immediate, personalized feedback (4, 5).

With the emergence of AI technologies, there is growing recognition of their potential to address these challenges and enhance surgical education quality. AI technologies, including machine learning, natural language processing, and computer vision, can analyze vast surgical datasets, simulate complex surgical scenarios, and provide real-time feedback to learners. These capabilities not only make surgical training more standardized but also help tailor learning paths according to each resident's individual needs and progress (6–8).

While research publications on AI applications in surgical education have notably increased, a comprehensive understanding of the field—including key trends, influential contributors, and evolving themes—remains lacking. Bibliometric analysis, as a powerful tool for identifying patterns and gaps through quantification and visualization of scientific literature, offers an effective framework for addressing this knowledge gap. By examining publication trends, author impact, institutional contributions, and keyword co-occurrence, bibliometric research can provide a holistic perspective on AI in surgical education research.

Therefore, this study aims to conduct a bibliometric analysis of research on AI in surgical education, covering the period from the field's inception to the present. We hope to provide a comprehensive overview of the current state of research in this field and offer insights that can guide future research and practice.

## Methodology

This study utilized the Web of Science Core Collection as the sole data source, with the search period ending on May 13, 2025. Web of Science is one of the most internationally recognized authoritative literature databases, with comprehensive index coverage, broad academic scope, and high-quality journals, making it suitable for conducting a high-quality bibliometric analysis of AI and surgical education.

The search was conducted using the following topic terms: “surgery” AND “education” AND “artificial intelligence”. Based on the search results, a total of 572 documents were retrieved, including 370 original articles and 12 highly cited papers, reflecting the growing interest in AI within surgical education. All search results were exported as plain text (.txt) format with full record and cited references, and then imported into R language for processing and analysis using the bibliometrix package (version 4.2). Inclusion criteria included: (1) Publications indexed in Web of Science Core Collection; (2) Topic terms containing “surgery” AND “education” AND “artificial intelligence”; (3) All document types; (4) Publication period from 1997 to May 13, 2025. Exclusion criteria included: (1) Duplicate records; (2) Non-English publications; (3) Records with incomplete metadata. The entire analysis followed the official bibliometrix workflow, including data import, cleaning, conversion, and analysis.

The data processing workflow included the following steps: First, duplicate records were identified and removed based on DOI and title matching algorithms. Second, author names and institutional affiliations were standardized to account for variations in spelling and formatting. Third, keyword fields were extracted and cleaned by removing punctuation inconsistencies and unifying term variations. Fourth, missing data fields were verified against the original Web of Science records. No manual content screening or quality assessment of individual articles was performed to maintain methodological consistency and ensure reproducibility of the analysis. All analytical code and processed datasets are available upon reasonable request.

The analysis content primarily included the following aspects: basic publication characteristic analysis (including annual publication volume, average annual publications, average citation count, annual growth rate, etc.); high-impact journal analysis (evaluating journals by publication volume, citation frequency, and Local H-index); author and institutional analysis (identifying highly productive authors, highly cited authors, collaboration network density, and co-authorship relationships); national/regional distribution and collaboration analysis (showing each country's output in this research field and the degree of international collaboration); highly cited paper analysis (identifying representative research with profound influence in this interdisciplinary field); keyword and topic analysis (identifying research hotspots through Author's Keywords and Keywords Plus, drawing Thematic Maps to evaluate the maturity and centrality of each topic); and citation network and co-citation analysis (identifying core literature clusters and academic community structures).

## Results

### General characteristics of the literature

As shown in Table 1, among the 572 documents (with 19,165 cited references), 3,228 authors participated in related research. The research began in 1997 and reached its current peak in 2025, showing a steep development trend. The annual growth rate was 18.39%, indicating the rapid development of this field.

**Table 1:** General information of the included publications.

Description	Results
Main information about data	
Time span	1997:2025
Sources (Journals, Books, etc.)	332
Documents	572
Annual Growth Rate %	18.39
Document Average Age	1.73
Average citations per doc	10.36
References	19,165
Document contents	
Keywords Plus (ID)	758
Author's Keywords (DE)	1,355
Authors	
Authors	3,228
Authors of single-authored docs	24
Authors collaboration	
Single-authored docs	25
Co-Authors per Doc	6.27
International co-authorships %	25.17
Document types	
article	370
article; book chapter	2
article; early access	30
article; proceedings paper	4
article; retracted publication	1
editorial material	11
letter	7
meeting	1
meeting abstract	1
proceedings paper	15
review	123
review; early access	7

This table presents comprehensive bibliometric data extracted from the web of science core collection. The 18.39% annual growth rate indicates the rapidly expanding interest in this field. The international co-authorship percentage (25.17%) reflects significant cross-border collaboration, while the document average age (1.73 years) highlights the recency of publications. The average citations per document (10.36) suggests moderate-to-high academic impact for this emerging interdisciplinary field. All 572 documents were included in the analysis.

A total of 332 journals published related research. International collaborative research accounted for 25.17%, indicating that cross-country research collaboration occupies an important position in this field. The average number of citations per document was 10.36, reflecting that the overall academic impact of the research is at a medium-high level.

### Annual publication trends & journal distribution

**Figure 1:**
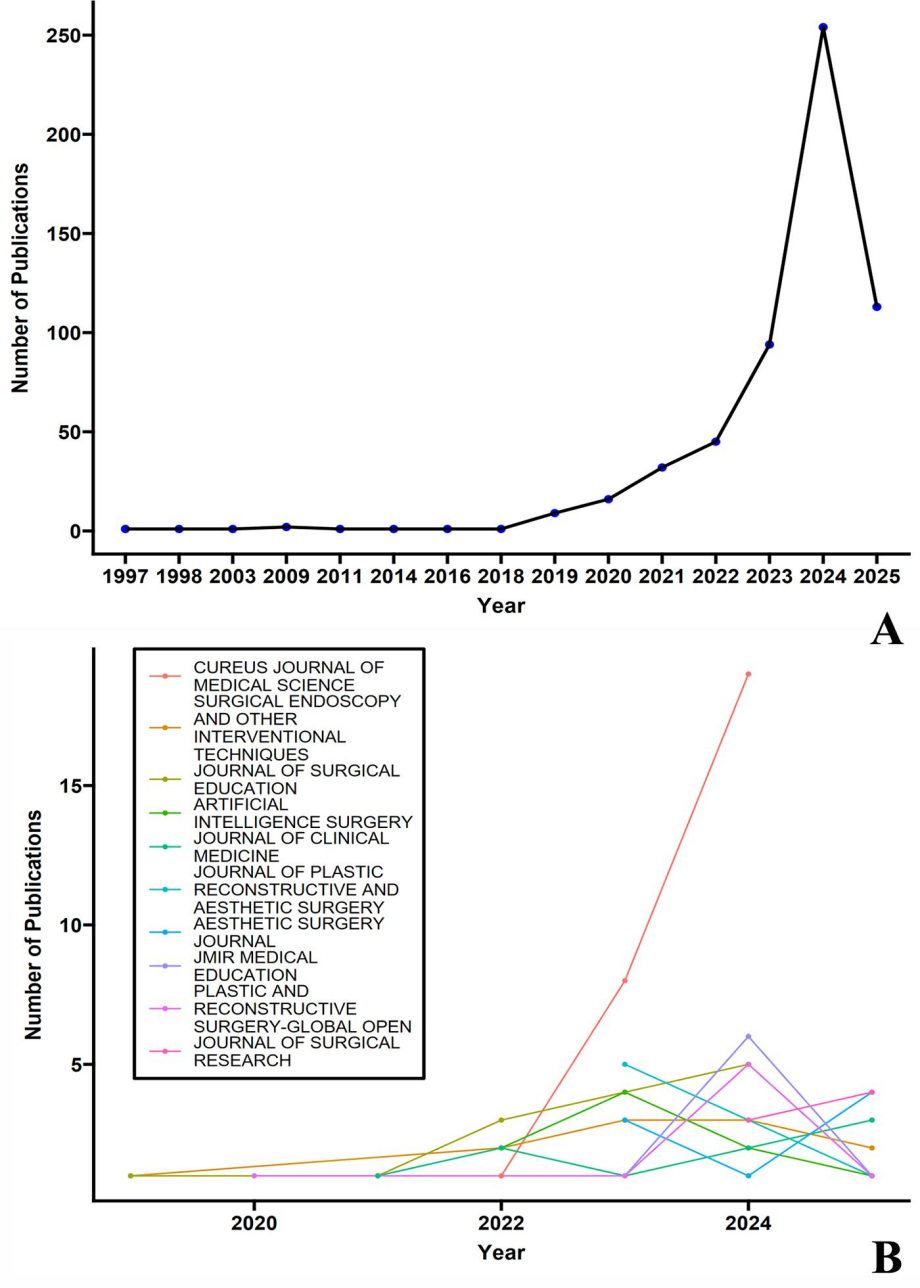
Publication trends of artificial intelligence in surgical education research (1997–2025). **(A)** Annual publication volume showing exponential growth from a single publication in 1997 to 254 publications in 2024. Data for 2025 is incomplete and represents only partial year results. **(B)** Annual publication trends of major journals contributing to this field, with Cureus Journal of Medical Science demonstrating particularly notable growth in recent years.

As shown in Figure 1A, the number of publications was 1 in 1997, increasing to an impressive 254 in 2024, demonstrating an exponential growth trend. It should be noted that the data for 2025 is incomplete and does not represent the full year's publication volume.

Figure 1B shows the annual publication trends of major journals, with the overall trend consistent with the total volume, increasing year by year. The growth of the Cureus Journal of Medical Science is particularly notable. This journal is currently indexed in ESCI (Emerging Sources Citation Index), with an impact factor of 1.0 in 2023. The journal has seen a significant increase in publications in recent years, with over 17,000 articles published in 2023, demonstrating its appeal in rapid publication and open access.

### Core journal analysis

**Figure 2:**
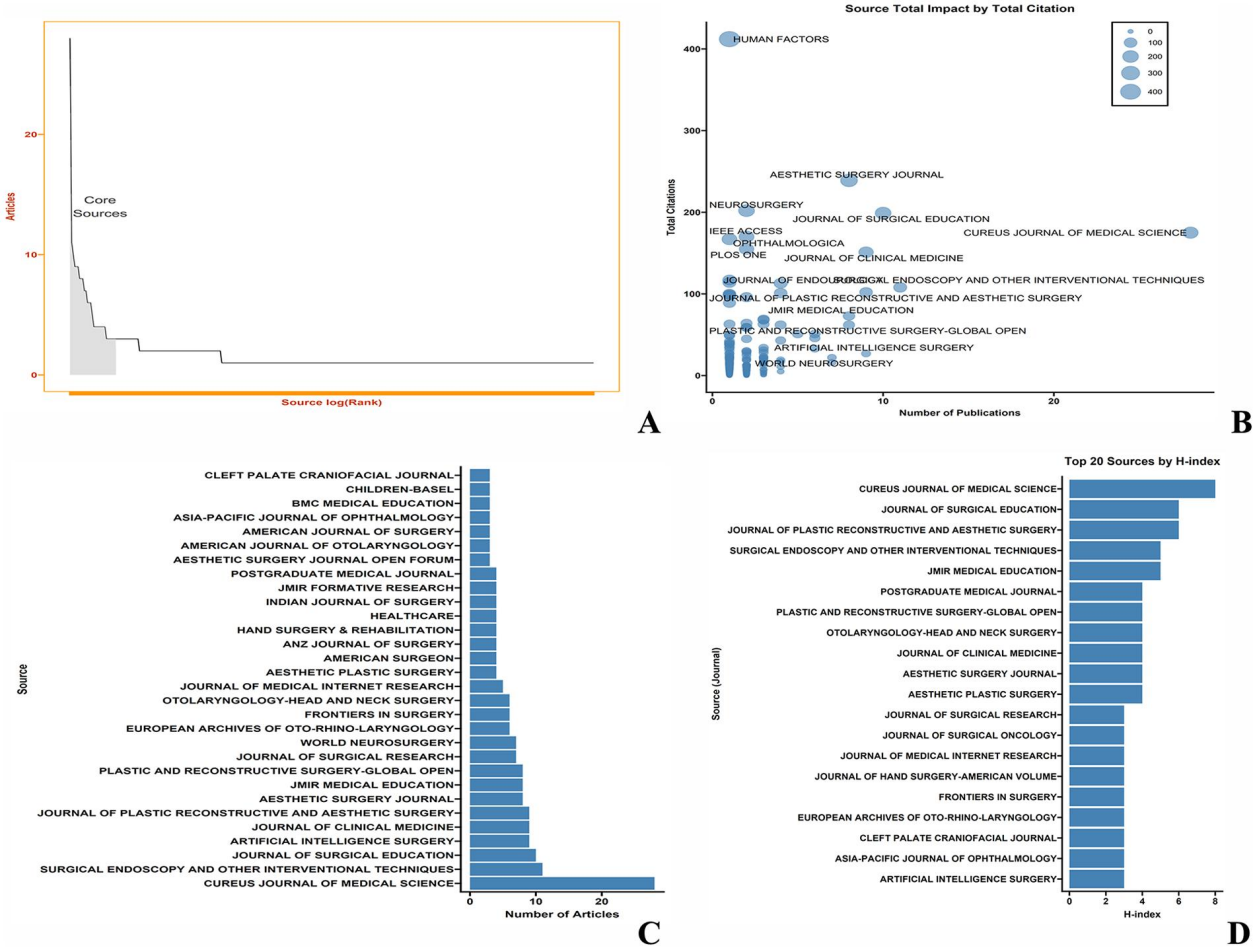
Core journal analysis in AI surgical education research. **(A)** Bradford's Law visualization demonstrating concentration of research publications in a small number of core journals. **(B)** Bubble chart displaying journal impact: horizontal axis represents publication count (right = higher count), vertical axis represents citation frequency (higher = more citations). **(C)** Top 30 core journals by publication volume, with Cureus Journal of Medical Science, Surgical Endoscopy and Other Interventional Techniques, and Journal of Surgical Education leading. **(D)** Top 20 journals ranked by H-index, highlighting Cureus Journal of Medical Science, Journal of Surgical Education, and Journal of Plastic Reconstructive and Aesthetic Surgery as most influential.

As shown in Figure 2A, research in this category follows Bradford's Law, meaning that most research is concentrated in a small number of core journals. Figure 2B uses bubble charts to display the number of publications and citation count of journals: the horizontal axis represents the number of publications, with more publications indicated by a position further to the right; the vertical axis represents the number of citations, with higher total citations indicated by a position higher up.

Among these, Human Factors has the highest total citations. This journal is published by SAGE Publications Inc., founded in 1958, and is the flagship journal of the Human Factors and Ergonomics Society. It is recognized in the academic community for its high-quality peer review and strict illustration standards. The significant increase in citations for this journal is primarily due to Sheridan's paper “Human-robot interaction: status and challenges” with 412 citations (1).

Figure 2C shows the top 30 core journals, with the top three being: CUREUS JOURNAL OF MEDICAL SCIENCE, SURGICAL ENDOSCOPY AND OTHER INTERVENTIONAL TECHNIQUES, AND JOURNAL OF Surgical Education. Figure 2D shows the top 20 journals ranked by H-index, with the top three including: CUREUS JOURNAL OF MEDICAL SCIENCE, JOURNAL OF SURGICAL EDUCATION, and JOURNAL OF PLASTIC RECONSTRUCTIVE AND AESTHETIC SURGERY. The H-index measures both the productivity and citation impact of publications within a specific research field.

### Key authors & institutions

Table 2 shows the most influential authors in this field. Raghav Gupta and Bryan Lim have the largest number of publications. Raghav Gupta is from the Icahn School of Medicine at Mount Sinai in the United States, while Bryan Lim is from Peninsula Health in Australia. It's worth noting that several authors who have collaborative relationships with Bryan Lim, including Richard J. Ross, Warren M Rozen, and Ishith Seth, also appear on the list of highly productive authors.

**Table 2:** Leading authors of the field.

Author	Articles	H_index	G_index	M_index	TC	NP	PY_start	Country	Affiliation	Sub-specialty
Raghav Gupta	6	4	6	1	120	6	2022	USA	Icahn School of Medicine at Mount Sinai	Urology
Bryan Lim	6	4	6	1.333	62	6	2023	Australia	Peninsula Health	Plastic and Reconstructive Surgery
Richard J. Ross	4	4	4	1.333	103	4	2023	Australia	Peninsula Health	Plastic and Reconstructive Surgery
Warren M Rozen	4	4	4	1.333	103	4	2023	Australia	Peninsula Health	Plastic and Reconstructive Surgery
Ishith Seth	6	4	6	1.333	132	6	2023	Australia	Peninsula Health	Plastic and Reconstructive Surgery
Neil L Dorward	3	3	3	0.6	41	3	2021	United Kingdom	University College London	Neurosurgery
Isabel Herzog	4	3	4	1	110	4	2023	USA	Rutgers New Jersey School of Medicine	Plastic Surgery
Andrew J Hung	4	3	3	0.375	185	3	2018	USA	Cedars-Sinai Medical Center	Robotic surgery
Danyal Z Khan	4	3	4	0.6	41	4	2021	United Kingdom	National Hospital for Neurology and Neurosurgery	Neurosurgery
Karl-Friedrich Kowalewski	4	3	3	0.429	65	3	2019	Germany	University of Heidelberg	General, Visceral, and Transplantation Surgery

This table identifies the most influential researchers in the field based on publication count, citation impact, and bibliometric indices. H_index indicates the number of papers with at least that many citations; G_index represents the highest number g where the top g articles received at least g^2^ citations; M_index is the H_index divided by years since first publication. TC, total citations; NP, number of publications; PY_start, first publication year in this field. Note the prominence of plastic and reconstructive surgery and neurosurgery specialties, and the collaborative network evident among Australian authors (Lim, Ross, Rozen, Seth).

From a disciplinary perspective, most authors come from plastic and reconstructive surgery, followed by neurosurgery. This reflects the high level of interest in AI-assisted technologies in these specialized fields, possibly because these surgical specialties involve complex anatomical structures and precise operations, creating a more urgent need for advanced educational tools (9).

**Figure 3:**
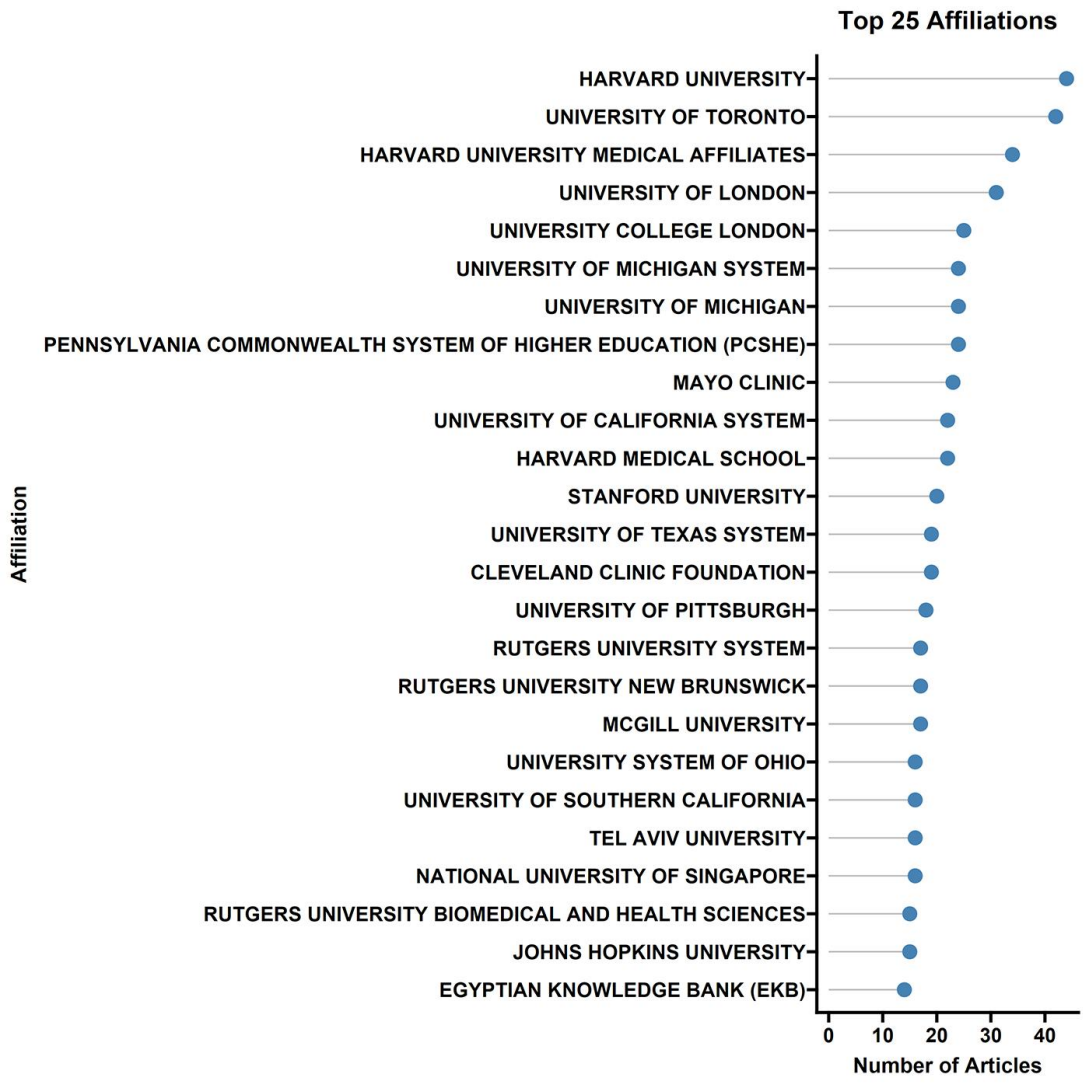
Top 25 affiliations contributing to AI in surgical education research. Lollipop chart displaying institutional output, with Harvard University, University of Toronto, Harvard University Medical Affiliates, University of London, and University College London as the top five contributing institutions. Note that institution names were not merged for this analysis, potentially affecting rankings where variations of institutional names exist.

Figure 3 shows the top 25 affiliations. The top five are: HARVARD UNIVERSITY, UNIVERSITY OF TORONTO, HARVARD UNIVERSITY MEDICAL AFFILIATES, UNIVERSITY OF LONDON, and UNIVERSITY COLLEGE LONDON. It should be noted that since the same institution may appear under different names in different documents, the rankings may contain slight errors. To respect the original data, this study did not process name merging.

### Highly cited publications analysis

Table 3 displays the ten most cited studies. Analysis of highly cited publications helps identify the most influential research that has shaped the development of AI applications in surgical education. Ranked first is Sheridan's paper “HUMAN-ROBOT INTERACTION: STATUS AND CHALLENGES” published in HUMAN FACTORS in 2016 (1). This study summarized the rapid development of the human-robot interaction (HRI) field, transitioning from primarily master-slave control in nuclear material handling to multi-functional robots with artificial intelligence capabilities. Current research focuses on human factors, medical applications, and autonomous control systems; although human-robot collaboration has been applied in many fields, truly effective universal robots remain to be validated.

**Table 3:** Top 10 most cited articles.

Authors	Year	Source	Title	Total citations
SHERIDAN TB	2016	HUMAN FACTORS	HUMAN-ROBOT INTERACTION: STATUS AND CHALLENGES	412
WONG TY;SABANAYAGAM C	2020	OPHTHALMOLOGICA	STRATEGIES TO TACKLE THE GLOBAL BURDEN OF DIABETIC RETINOPATHY: FROM EPIDEMIOLOGY TO ARTIFICIAL INTELLIGENCE	167
MIRCHI N;BISSONNETTE V;YILMAZ R;LEDWOS N;WINKLER-SCHWARTZ A;DEL MAESTRO RF	2020	PLOS ONE	THE VIRTUAL OPERATIVE ASSISTANT: AN EXPLAINABLE ARTIFICIAL INTELLIGENCE TOOL FOR SIMULATION-BASED TRAINING IN SURGERY AND MEDICINE	146
BHAT JR;ALQAHTANI SA	2021	IEEE ACCESS	6G ECOSYSTEM: CURRENT STATUS AND FUTURE PERSPECTIVE	143
WINKLER-SCHWARTZ A;BISSONNETTE V;MIRCHI N;PONNUDURAI N;YILMAZ R;LEDWOS N;SIYAR S;AZARNOUSH H;KARLIK B;DEL MAESTRO RF	2019	JOURNAL OF SURGICAL EDUCATION	ARTIFICIAL INTELLIGENCE IN MEDICAL EDUCATION: BEST PRACTICES USING MACHINE LEARNING TO ASSESS SURGICAL EXPERTISE IN VIRTUAL REALITY SIMULATION	126
HUNG AJ;CHEN J;CHE ZP;NILANON T;JARC A;TITUS M;OH PJ;GILL IS;LIU Y	2018	JOURNAL OF ENDOUROLOGY	UTILIZING MACHINE LEARNING AND AUTOMATED PERFORMANCE METRICS TO EVALUATE ROBOT-ASSISTED RADICAL PROSTATECTOMY PERFORMANCE AND PREDICT OUTCOMES	117
GHAEDNIA H;FOURMAN MS;LANS A;DETELS K;DIJKSTRA H;LLOYD S;SWEENEY A;OOSTERHOFF JHF;SCHWAB JH	2021	SPINE JOURNAL	AUGMENTED AND VIRTUAL REALITY IN SPINE SURGERY, CURRENT APPLICATIONS AND FUTURE POTENTIALS	114
SPICER MA;APUZZO MLJ	2003	NEUROSURGERY	VIRTUAL REALITY SURGERY: NEUROSURGERY AND THE CONTEMPORARY LANDSCAPE	107
LIU PR;LU L;ZHANG JY;HUO TT;LIU SX;YE ZW	2021	CURRENT MEDICAL SCIENCE	APPLICATION OF ARTIFICIAL INTELLIGENCE IN MEDICINE: AN OVERVIEW	100
OH N;CHOI GS;LEE WY	2023	ANNALS OF SURGICAL TREATMENT AND RESEARCH	CHATGPT GOES TO THE OPERATING ROOM: EVALUATING GPT-4 PERFORMANCE AND ITS POTENTIAL IN SURGICAL EDUCATION AND TRAINING IN THE ERA OF LARGE LANGUAGE MODELS	99

This table presents the ten most cited articles in the field, demonstrating the research impact of various AI applications in surgical education and training. The high citation counts for human-robot interaction (1) and AI-based simulation training (3) highlight their foundational importance in the field. Recent publications on large language models (13) indicate emerging research interest in applying generative AI to surgical education. The distribution of publication years (2003–2023) reflects the evolution from virtual reality simulation to advanced AI applications in this rapidly developing field. Highly cited publications reflect their influence on the indexed literature and may include studies with broader relevance to AI applications in medical and surgical education contexts.

Ranked second is Wong's 2020 paper in OPHTHALMOLOGICA: “STRATEGIES TO TACKLE THE GLOBAL BURDEN OF DIABETIC RETINOPATHY: FROM EPIDEMIOLOGY TO ARTIFICIAL INTELLIGENCE” (2). This study points out that telemedicine screening for diabetic retinopathy incorporating AI has the potential to improve screening efficiency and coverage rates in low- and middle-income countries.

Ranked third is Mirchi's 2020 paper in PLOS ONE: “THE VIRTUAL OPERATIVE ASSISTANT: AN EXPLAINABLE ARTIFICIAL INTELLIGENCE TOOL FOR SIMULATION-BASED TRAINING IN SURGERY AND MEDICINE” (3). This study developed the Virtual Operative Assistant, an explainable AI education platform for surgical simulation training. The experiment used supporting vector machines for performance indicator training and validation in neurosurgical simulation surgery, successfully distinguishing between experienced and novice learners with 92% accuracy, 82% specificity, and 100% sensitivity.

### Network analysis

**Figure 4:**
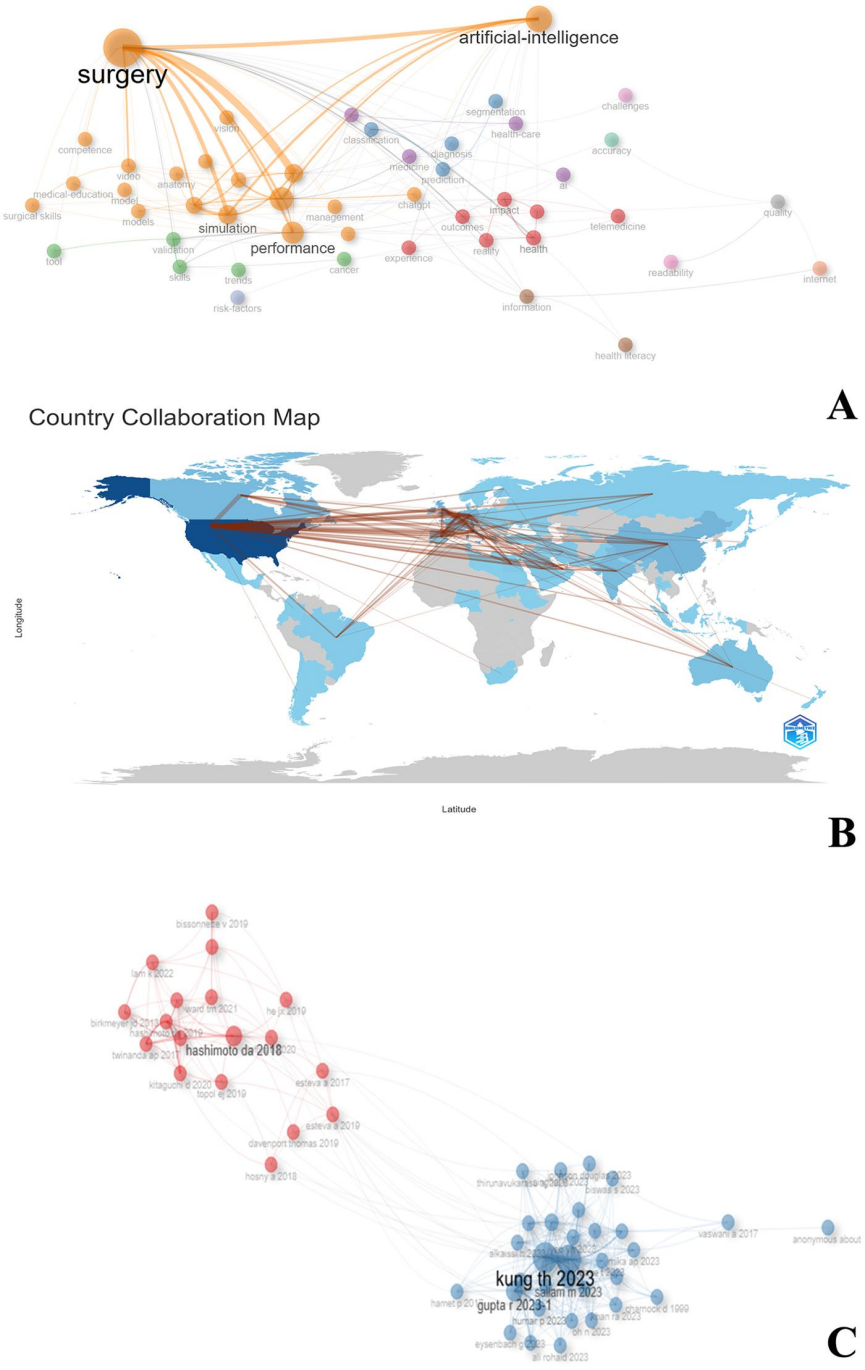
Network analysis of AI in surgical education research. **(A)** Keyword co-occurrence network showing Surgery and Artificial Intelligence as core research topics, closely connected with concepts like Simulation and Performance, illustrating key focus areas in the field. **(B)** Country collaboration map depicting international research partnerships, with the United States at the core position and strong participation from the United Kingdom, Germany, Australia, and other developed nations. **(C)** Co-citation network revealing two main research clusters: one centered on Hashimoto's 2018 paper “Artificial Intelligence in Surgery: Promises and Perils” and another around Kung's 2023 paper “Performance of ChatGPT on USMLE,” representing two key research directions in the field.

Figure 4 shows the results of three types of network structure analysis:

- Co-occurrence Network: Keyword analysis shows that Surgery and Artificial Intelligence are the current core research topics, closely connected with keywords such as Simulation and Performance, reflecting the importance of simulation training and performance evaluation in surgical AI applications.
- Country Collaboration Map: It shows the collaborative relationships between major global economies in this research field, with the United States at the core position, and the United Kingdom, Germany, Australia, etc., also having high participation, revealing an international collaboration model led by developed countries. This reflects the leading position of these countries in medical AI research and their sound research infrastructure and collaborative traditions.
- Co-citation Network: Indicates two main research clusters. One centers on “Artificial Intelligence in Surgery: Promises and Perils” published by Daniel A Hashimoto in 2018 in Annals of Surgery, systematically discussing the current status and prospects of machine learning, artificial neural networks, natural language processing, and computer vision in surgery (4); the other cluster centers on “Performance of ChatGPT on USMLE” published by Tiffany H Kung in 2023 in PLOS Digital Health, demonstrating the application potential of ChatGPT in standardized medical exams and suggesting the value of large language models in medical education (10). These two clusters represent two key research directions: AI applications in surgery and medical education evaluation.

### Thematic map analysis

**Figure 5:**
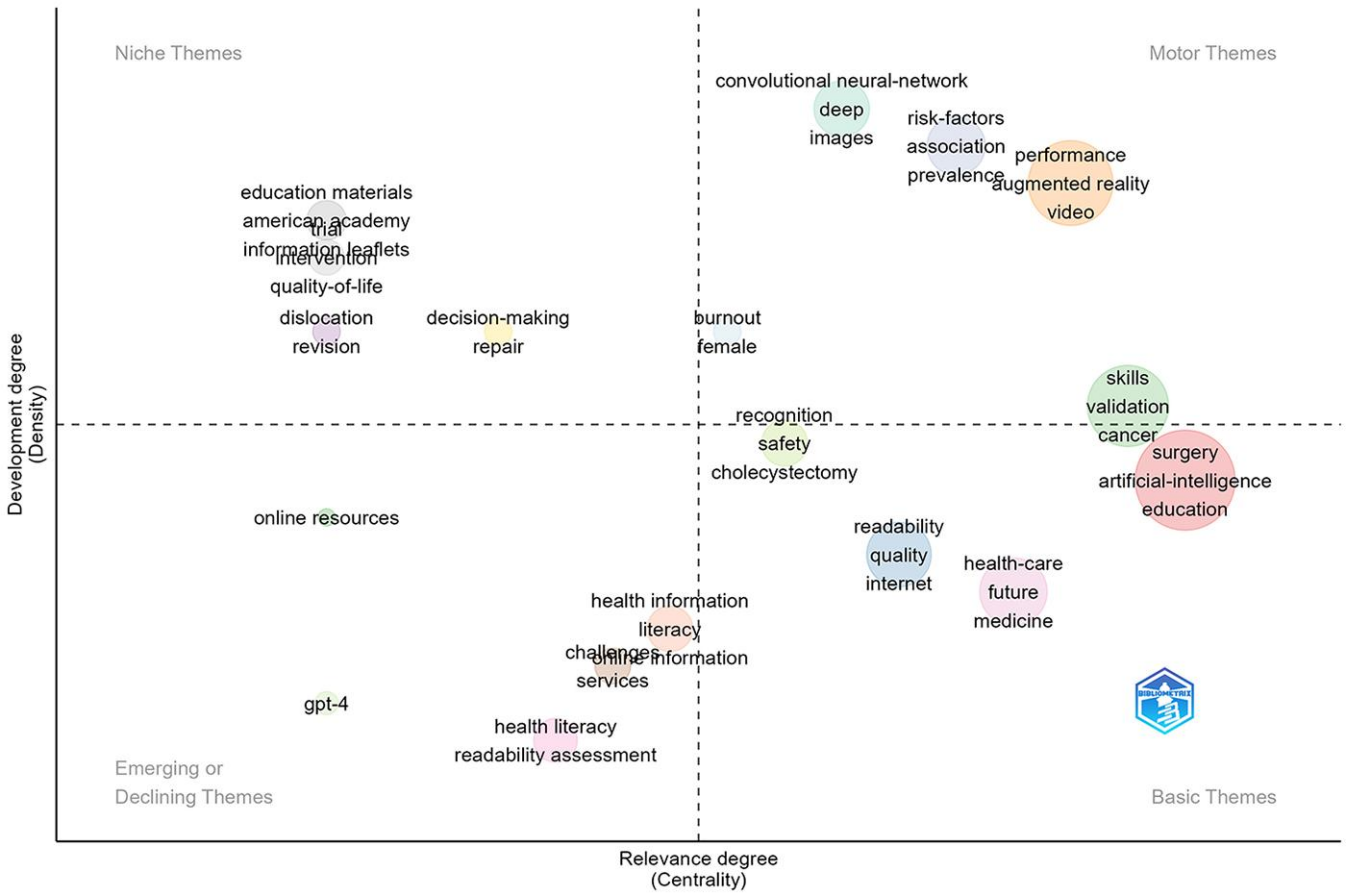
Thematic map of research topics in AI surgical education. Quadrant visualization of research themes based on development degree (density, *y*-axis) and relevance degree (centrality, *x*-axis). Upper right quadrant (motor themes) includes mature and dynamic research areas such as augmented reality/video/performance, convolutional neural network/deep learning/images, skills/validation, and the central surgery/artificial intelligence/education cluster. Upper left quadrant (Niche themes) contains specialized topics with depth but limited influence, including education materials, information leaflets, quality-of-life, dislocation/revision, and decision-making/repair. lower left quadrant (emerging or declining themes) features exploratory topics such as GPT-4, online resources, health literacy/readability assessment, and health information/challenges. Lower right quadrant (basic themes) shows foundational concepts with high centrality but lower density, including readability/quality/internet, health-care/future/medicine, and recognition/safety/cholecystectomy.

Figure 5 is a thematic map used to show the development maturity (Density) and academic importance (Centrality) between research topics. This map divides topics into four categories:

**Motor Themes (upper right quadrant)**: Including Augmented Reality, Video, Performance, Convolutional Neural Network/Deep Learning/Images, Skills/Validation, and the central Surgery/Artificial Intelligence/Education cluster, representing research directions that are currently mature and highly dynamic. Augmented reality technology is increasingly becoming an important tool for surgical education.

**Niche Themes (upper left quadrant)**: Such as Education Materials, Information Leaflets, Quality-of-life, Intervention, Dislocation/Revision, Decision-making/Repair, with relatively deep research but limited scope of influence. Although these topics are highly specialized, they have not yet formed a broad impact in the current research ecosystem and may require more interdisciplinary integration to expand their application scope.

**Emerging or Declining Themes (lower left quadrant)**: Including GPT-4, Online Resources, Health Literacy, Readability Assessment, Health Information, Challenges, and Online Information Services, belonging to exploratory topics that are not yet mature, or possibly increasingly marginalized directions. The emergence of GPT-4 indicates its emerging research value in medical education.

**Basic Themes (lower right quadrant)**: Covering Readability/Quality/Internet, Health-care/Future/Medicine, and Recognition/Safety/Cholecystectomy near this quadrant boundary, and other foundational issues, with relatively low research density but playing a key role in constructing the research system and promoting interdisciplinary integration. Although these concepts are fundamental, they are critical for understanding the overall application of AI in surgical education.

## Discussion

Through bibliometric methods, this study systematically analyzed the research status and development trends of artificial intelligence (AI) in surgical education. The following sections discuss in depth from the perspectives of main findings, application potential of AI in the surgical field, importance of international collaboration, future research directions, and limitations of the study.

## Primary research findings

This study found that research on AI in surgical education is showing a rapid growth trend, with an annual growth rate of 18.39%, indicating that this field is receiving widespread attention from global researchers. Research topics cover a broad range from basic technology development to clinical applications, with augmented reality, video, and performance already becoming mature research directions (motor themes), while GPT-4 and other large language models represent emerging research hotspots. The rapid growth in publications on AI in surgical education, particularly from 2019 onwards, can be attributed to several converging factors. First, advances in machine learning algorithms and deep learning architectures have made AI applications more feasible and accurate for surgical skill assessment and training. Second, the proliferation of affordable computing resources and cloud-based platforms has lowered the barrier to entry for AI research in medical institutions. Third, there is growing recognition of the limitations of traditional apprenticeship models and the need for objective, scalable assessment methods in surgical education.

In terms of research institution distribution, Harvard University and University of Toronto and other top universities occupy leading positions, reflecting the key role of high-level research institutions in promoting the integration of AI and surgical education. The country collaboration network analysis shows that the United States is at the center of the global collaboration network, forming close research collaborative relationships with the United Kingdom, Germany, Australia, and other developed countries. However, the participation of developing countries in this field is relatively low, indicating a significant “digital divide” phenomenon.

In terms of journal distribution, the research follows Bradford's Law (11), meaning that most documents are published in a few core journals. Among them, “Cureus Journal of Medical Science”, “Journal of Surgical Education”, and other specialized journals have become the main publication platforms in this field. Highly cited document analysis shows that AI applications in the surgical field are mainly concentrated in surgical simulation training, skill evaluation, and decision support systems, and these studies have laid the theoretical foundation for the in-depth application of AI technology in surgical education.

In addition, keyword co-occurrence analysis shows that “Surgery”, “Artificial Intelligence”, “Simulation”, “Performance”, and other concepts constitute the core terminology system of this field, while thematic map analysis further reveals the dynamic relationship between mature themes (motor themes) such as augmented reality, video, and performance, and emerging themes like GPT-4. These findings not only depict the overall picture of AI research in surgical education but also provide important reference for grasping the future development direction of this field.

## Application potential of AI in the surgical field

Based on existing literature and the results of this study, AI demonstrates enormous application potential in the surgical field, especially in surgical education.

First, AI plays an important role in surgical skill evaluation. Through video analysis and automatic evaluation technology, AI can objectively and accurately assess the skill level of surgical physicians. This evaluation method not only reduces human bias but can also provide personalized feedback and recommendations to help surgeons better improve their skills. At the same time, motion tracking and biometric recognition technologies also provide real-time feedback and personalized guidance for surgical physicians' skill improvement. For example, by tracking a surgeon's surgical movements, AI can analyze the accuracy and efficiency of their operations, thereby providing targeted improvement suggestions (5).

Second, the personalized applications of AI in surgical education are also worth noting. Traditional surgical education often adopts a “one-size-fits-all” teaching approach, which is difficult to meet the personalized needs of each resident. AI technology, however, can tailor individualized learning paths and teaching resources based on each resident's skill level, learning style, and interest preferences. For example, through AI technology, the surgical skills and experience of experienced surgeons can be transformed into learnable data models, providing personalized learning recommendations and feedback for each resident. Additionally, virtual reality simulation systems can create highly realistic surgical scenarios to help surgical physicians practice repeatedly in simulated environments, improving surgical proficiency and ability to handle complex situations.

AI also has significant advantages in surgical education material improvement and remote education. Traditional surgical education materials are often primarily text and images, making it difficult to intuitively display the surgical process and details. AI technology, however, can automatically extract and classify key segments from surgical videos to provide clear, structured teaching materials for surgical education. These teaching materials not only help residents better understand the surgical process and techniques but also serve as important references for teaching evaluation and feedback. At the same time, the combination of AI and communication technology has also promoted the development of remote surgical teaching. Through AI platforms, surgeons from different regions and different hospitals can conduct real-time exchanges and collaboration, jointly solving problems during surgery, improving the overall medical level (12).

## International collaboration and development gaps

Our analysis revealed significant disparities in AI research for surgical education. Developed nations, particularly the United States, United Kingdom, and Germany, dominate the research landscape due to their robust infrastructure, funding, and innovation capabilities. This creates a concerning “digital divide” as developing countries show limited participation in this field.

To address this gap, increased international collaboration and technology transfer are essential. Research institutions from developed countries should establish partnerships with developing regions, while open-source solutions and locally appropriate AI technologies could help democratize access. These efforts are crucial for ensuring that advances in surgical AI education benefit medical training globally rather than exacerbating existing healthcare inequalities.

## Future research directions

Future research should prioritize several key areas to advance AI in surgical education:

**Personalized Learning Systems**: Develop AI tools that adapt to individual learning styles, skill levels, and specialty-specific requirements.

**AI-Traditional Teaching Integration**: Create hybrid models that combine AI's analytical capabilities with the irreplaceable human elements of surgical mentorship.

**Explainability and Ethics**: Enhance transparency in AI decision-making processes while addressing data privacy, algorithmic bias, and responsibility attribution.

**Emerging Technology Integration**: Combine AI with technologies like 5G, IoT, and edge computing to create responsive surgical training environments that provide real-time feedback and immersive learning experiences.

These directions should be pursued with careful attention to ethical implementation, ensuring AI augments rather than replaces the human elements central to surgical education.

## Research limitations

This study has several limitations. First, our data is limited to the Web of Science Core Collection, which may not capture all relevant publications indexed in other databases such as Scopus, PubMed, or regional databases. Second, citation lag bias may affect our analysis, as recent publications have had less time to accumulate citations compared to older works. Third, our search was limited to English-language publications, which may introduce language bias and underrepresent research published in other languages. Fourth, the bibliometric approach itself has inherent methodological constraints—it relies on quantitative metrics that may not fully capture research quality or impact. Fifth, we did not perform manual data cleaning or refinement of the dataset after the initial automated search, which may have resulted in the inclusion of some peripherally relevant publications. However, this approach was chosen to maintain methodological consistency and reproducibility of the analysis.

## Conclusion

This bibliometric analysis provides a comprehensive overview of AI research in surgical education spanning nearly three decades (1997–2025). Our findings reveal several key patterns in this emerging field.

First, the field has experienced exponential growth, with publications increasing from a single article in 1997 to 254 in 2024, representing an annual growth rate of 18.39%. Notably, substantial and sustained research activity became evident from 2019 onward, reflecting the maturation of AI technologies and growing recognition of their potential in surgical training.

Second, geographic disparities in research output are pronounced. The United States dominates the field, followed by the United Kingdom and Germany, with Harvard University and University of Toronto emerging as the most productive institutions. International collaboration accounts for 25.17% of publications, indicating opportunities for expanded cross-border partnerships to share expertise and resources.

Third, the research landscape is characterized by diverse AI applications. Thematic analysis reveals that augmented reality, video, and performance represent mature research areas (motor themes), while emerging topics such as large language models (GPT-4) and online educational resources remain in exploratory stages. The field demonstrates integration across multiple surgical specialties, with plastic surgery and neurosurgery showing particularly strong engagement.

Fourth, publication patterns follow Bradford's Law, with research concentrated in a core set of journals including Cureus Journal of Medical Science, Surgical Endoscopy, and Journal of Surgical Education. Human Factors emerges as the most highly cited journal, reflecting the importance of human-machine interaction principles in this field.

These findings highlight both the dynamism and challenges of AI integration in surgical education. Future research should prioritize developing personalized learning systems, enhancing international collaboration to address global disparities, and ensuring ethical implementation of AI technologies while preserving the essential human elements of surgical mentorship.

## Data Availability

The original contributions presented in the study are included in the article/Supplementary Material, further inquiries can be directed to the corresponding author.
